# Influence of Stainless Steel Mesh Size and Universal Adhesive Primer on Flexural Strength of Repaired PMMA Denture Base Resin

**DOI:** 10.1002/cre2.70127

**Published:** 2025-04-07

**Authors:** Gray H. Li, Vidya Mudaliar, Andrew B. Cameron, John M. Aarts, Joanne J. E. Choi

**Affiliations:** ^1^ Sir John Walsh Research Institute, Faculty of Dentistry University of Otago Dunedin New Zealand; ^2^ Department of Oral Rehabilitation, School of Dentistry and Oral Health, College of Medicine Nursing and Health Science Fiji National University Suva Fiji; ^3^ School of Medicine and Dentistry Griffith University Gold Coast Queensland Australia

**Keywords:** denture reinforcement, denture repair, mesh reinforcement, metal adhesive primer, stainless steel mesh

## Abstract

**Objectives:**

To evaluate the effect of the size of stainless steel mesh and universal adhesive primer on the flexural strength of repaired polymethylmethacrylate (PMMA) denture base resin.

**Materials and Methods:**

A total of 120 heat‐cured PMMA specimens with dimensions of 5 × 50 × 30 mm were prepared and repaired with two different sizes of stainless steel mesh reinforcement, one group with med‐fine mesh (size 0.42 mm^2^) and the second group with fine mesh (size 0.09 mm^2^). One subgroup was primed with a universal adhesive primer (Scotchbond Universal). Half of the specimens from each subgroup were subjected to artificial aging. The flexural strength was obtained by three‐point bend testing. Data were statistically analyzed using ANOVA and post hoc analysis (SPSS V28). The probability of failure was calculated using Weibull analysis. Scanning electron microscopy analysis was used to identify the mode of failure.

**Results:**

A significantly higher mean flexural strength (*p* < 0.05) was recorded in primed groups non‐thermocycled with fine mesh (174.80 ± 50.27 MPa) and medium mesh (160.87 ± 41.50 MPa) compared to non‐primed groups. Non‐primed specimens with fine mesh exhibited the highest Weibull modulus (5.86), whereas that of primed medium mesh had the lowest Weibull modulus (2.64). Adhesive failure was identified at the interface of the stainless steel mesh and the self‐cure acrylic resin.

**Conclusion:**

Application of the universal adhesive primer to both mid‐fine and fine stainless steel mesh significantly improved the flexural strength of the repaired PMMA heat‐cured acrylic resin, and reinforcement with primed fine stainless steel mesh resulted in significantly higher flexural strength of repaired PMMA heat‐cured an acrylic resin.

## 1 Introduction

Polymethyl methacrylate (PMMA) is a commonly used material in the fabrication of complete dentures due to its aesthetic pink color and low water solubility and toxicity (Andreescu et al. 2018). Conversely, mechanical properties such as insufficient surface hardness, low fracture toughness, and low strength do not enable the PMMA material to be sufficient for long‐term clinical performance over 5–10 years (Taylor et al. 2021). Under cyclic loading from mastication and speech, fatigue failure can occur in PMMA material, which gives rise to the problem of a high fracture incidence of PMMA dentures (Alhareb et al. 2017; Beyli and Von Fraunhofer 1981). As a result, denture repair has become common practice (Seó et al. 2007). Self‐cure acrylic resin is often the choice of material in PMMA denture repair as it is economical, and the repair procedure is simple and less time‐consuming (Rached et al. 2004; AlQahtani and Haralur 2020). However, several study results have revealed a significant reduction of flexural strength in heat‐cure acrylic resin after it has been repaired with self‐cure acrylic resin (Heidari et al. 2015; Polyzois et al. 2001; Nagai et al. 2001; Arioli Filho et al. 2011). To facilitate the repair, reinforcement materials such as stainless steel mesh, glass fiber, and polyester fiber can be incorporated to increase the overall flexural strength of the repair (Minami et al. 2005). The use of stainless steel mesh in PMMA acrylic resin repairs has been reported to have the highest flexural strength (Anne et al. 2017; Ardakani et al. 2021). However, two previous studies have reported an insufficient micromechanical interaction of the stainless steel mesh and PMMA resulting in low flexural strength (Somani et al. 2019; Fonseca et al. 2015). A mechanical approach using microblasting is available to improve the alloy‐polymer flexural strength. Microblasting with aluminum oxide can increase the surface area of the alloy and facilitate micromechanical bonding (Kalra et al. 2015). However, discoloration of the alloy could occur after microblasting as well as potential plastic deformation in the microstructure of the alloy and a decrease in microhardness (Multigner et al. 2009). The study results of Goracci et al. (2019) showed that a chemical approach using universal adhesive primers improved the alloy‐polymer bonding significantly between the CAD/CAM PMMA blocks and stainless steel brackets. The study results further revealed that shear bond strength in specimens with application of Scotchbond Universal Adhesive and Assure Plus was significantly higher than in the group with Transbond XT Primer application (Goracci et al. 2019). It has been suggested that the chemical approach is more practical since the bond strength is obtained by a phosphoric acid monomer and silane in the adhesive primer chemically reacting with the metal oxide of stainless steel and binding to acrylic (Kim et al. 2009).

There are very few studies that have evaluated the flexural strength of PMMA acrylic resin that has been repaired using chemically pretreated stainless steel mesh. Additionally, none of these studies evaluated the influence of the size of the mesh on the flexural strength of repaired PMMA heat‐cured acrylic resin. Therefore, the aim of this study was to investigate the influence on flexural strength of PMMA heat cure repaired acrylic resin reinforced with stainless steel mesh with two different mesh sizes (medium‐fine [med‐fine] and fine). The impact of the application of an adhesive primer on stainless steel meshes and artificial aging on the flexural strength of PMMA heat‐cured acrylic resin was also evaluated. The null hypothesis was that there is no significant influence on the flexural strength of repaired PMMA heat‐cured acrylic resin reinforced with med‐fine and fine stainless steel mesh primed with adhesive and artificial aging.

## 2 Materials and Methods

### 2.1 Specimen Fabrication

**Figure 1:**
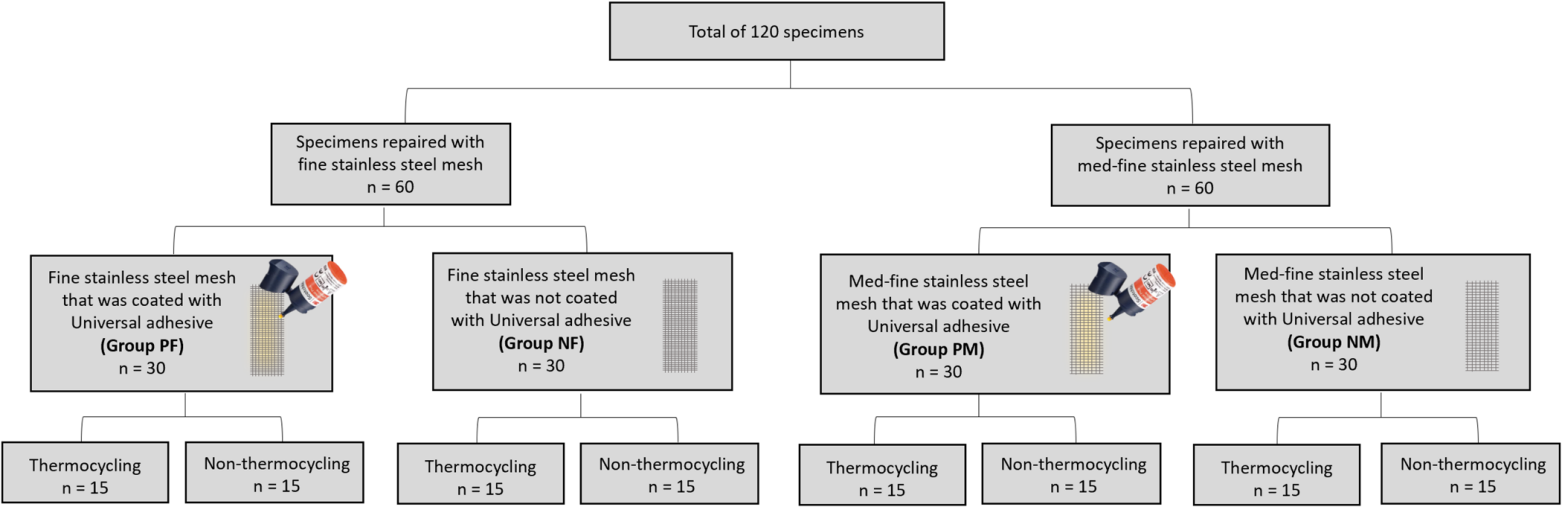
Specimen test groups: NF—non‐primed fine stainless steel mesh, ^P^F—primed fine stainless steel mesh, NM—non‐primed med‐fine stainless steel mesh, PM—primed med‐fine stainless steel mesh, NTC—non‐thermocycling, TC—thermocycling.

A total of 120 specimens were fabricated in dimensions of 5 × 10 × 30 mm (height × width × length). The testing protocol and specimen dimensions were based on a previous study (Li et al. 2021). The sample size calculation was based on this previous experiment with the software G*Power v3.0.10 (Heinrich‐Heine‐Universität Düsseldorf) to calculate the sample size. The calculation showed that 15 specimens per group are required. The 120 specimens were divided initially into two groups; one group of specimens was reinforced with fine stainless steel mesh (*n* = 60), and the other group was reinforced with med‐fine stainless steel mesh (*n* = 60). The specimen in the stainless steel mesh and med‐fine stainless steel mesh groups were further divided into two subgroups (*n* = 30); one group was primed with Universal Plus Adhesive primer (3M, Scotchbond) (Table 1), and the other group of the specimens was not primed (Figure 1). The specimens that were reinforced with non‐primed fine mesh were labeled as Group NF. The specimens that were reinforced with primed fine mesh were labeled as Group PF. The specimens that were reinforced with non‐primed med‐fine mesh were labeled as Group NM. The specimens that were reinforced with primed med‐fine mesh were labeled as Group PM.

**Table 1:** PMMA materials and stainless steel meshes included in the study.

Material	Name/Brand	Manufacturer	Composition
Denture Base Acrylics (heat‐cure acrylic resin)	Vertex Rapid Simplified	Vertex‐Dental B. V., Zeist, Netherlands	Methyl methacrylate > 95%, crosslinker < 5%, accelerator < 1%
Denture Base Acrylics (self‐cure acrylic resin)	Vertex CastaPress	Vertex‐Dental B. V., Zeist, Netherlands	Methyl methacrylate > 95%, crosslinker < 5%, accelerator < 1%, UV absorber < 1%
Med‐fine Stainless Steel Mesh (size: 0.4225 mm^2^, thickness: 0.2 mm)	Keystone Industries	Keystone Industries, Singen, Germany	Type 304 austenitic stainless steel
Fine Stainless Steel Mesh (size: 0.0992 mm^2^, thickness: 0.2 mm)	Dentaurum	Dentaurum, Ispringen, Germany	Type 304 austenitic stainless steel
Universal Plus Adhesive primer	3M Scotchbond	3M Deutschland GmbH, Germany	MDP phosphate monomer, 2‐hydroxyethyl methacrylate (HEMA), 3M Vitrebond copolymer, filler, ethanol/water, initiators, silane, dimethacrylate resins

**Figure 2:**
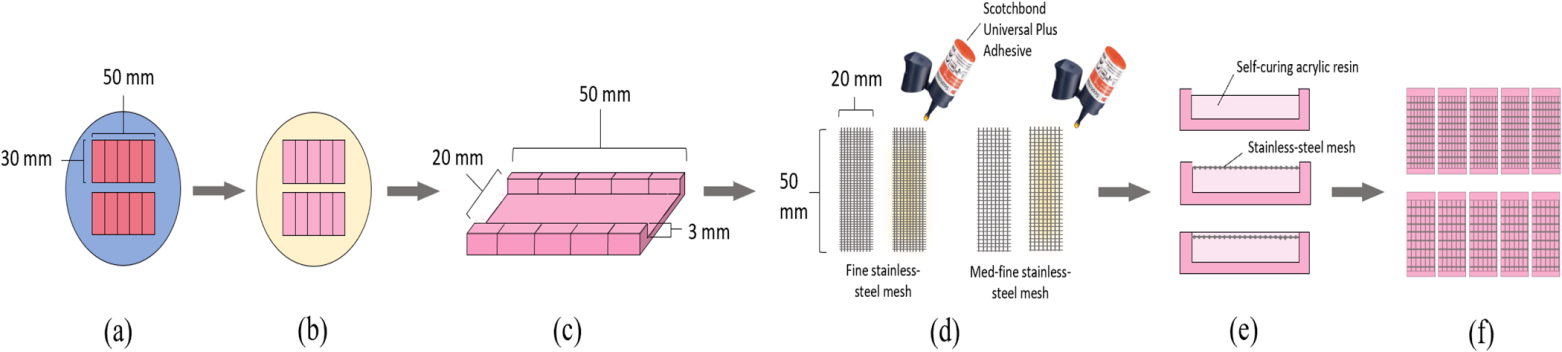
Sequence of specimen preparation: (a) wax specimen mold, (b) imbedding of wax in stone, (c) preparation of blocks for repairing, (d) stainless steel mesh preparation, (e) embedding of stainless steel mesh in acrylic, (f) top view of embedded mesh in acrylic.

The denture acrylic used for the specimens in this study was a heat‐cure denture base material (Vertex Rapid Simplified, Netherlands) (Table 1). The wax specimens were fabricated using a silicone mold (Figure 2a). The wax was melted and poured into the silicone mold and left on the bench for 30 min and then embedded in metal flasks using dental stones. The metal flasks were placed in a boil‐out unit (Wapo‐Ex 12 II, Wassermann, Germany) to soften the wax, and then the flasks were opened to allow the wax to be thoroughly flushed out with hot water. The acrylic resin was mixed following the correct mixing ratio provided by the manufacturer. The acrylic resin was packed into the flask and then cured in a polymerizing unit (Acrydig 12, Manfredi, Italy) at a temperature of 100°C for 30 min. Once the flasks cooled to room temperature, the acrylic specimens were removed from the stone (Figure 2b). A polishing machine (TegraPol‐21, Struers, Denmark) with 120 grit sandpaper at a speed of 300 rpm was used to achieve the intended dimension of the acrylic blocks. A customized three‐axis drill press machine (DP‐200B, Tooline, China) was employed to mill a repair site in a dimension of 3 × 50 × 20 mm for each acrylic resin block (Figure 2c). Following this, an automatic cutting machine (Accutom‐50, Struers, Denmark) was used to cut the blocks in half to simulate denture fracture before repairing them.

### 2.2 Specimen Repairing

The fine stainless steel mesh and the med‐fine stainless steel mesh samples were cut to the same dimensions as the repair site created in the acrylic block. Half of the fine mesh pieces (*n* = 30) and half of the med‐fine mesh pieces (*n* = 30) were smeared with a thin layer of adhesive primer (Universal Plus adhesive primer, 3M Scotchbond, Germany) and air‐dried for 20 s (Figure 2d). The remaining stainless steel mesh samples were not coated with the adhesive primer. The acrylic resin blocks were divided into four groups: NF group (*n* = 30) was repaired with non‐primed fine stainless steel mesh, PF group (*n* = 30) was repaired with primed fine stainless steel mesh, NM group (*n* = 30) was repaired with non‐primed mid‐fine stainless steel mesh, and PM group (*n* = 30) was repaired with primed mid‐fine stainless steel mesh. The acrylic resin blocks were held within a silicone putty mold during the repair process. The self‐curing acrylic resin (Vertex Castapress, Netherlands) was mixed following the manufacturer's instructions and poured in a free‐flowing state to fill ¾ of the repair space. To allow for the stainless steel mesh to be embedded in the tension zone, the stainless steel mesh was then carefully placed on top of the self‐cure acrylic resin using a tweezer, and another layer of the self‐cure acrylic resin was poured to cover the stainless steel mesh (Figure 2e). The width (20 mm) and depth (3 mm) of the repair gap was controlled by using the three‐axis drill press machine to standardize the method. The self‐cure acrylic resin was then cured in a pressure polymerization unit (Palamat elite, Kulzer, USA) for 20 min at 55°C. After processing, five specimens were cut out from one acrylic block using the automatic cutting machine (Accutom‐50, Struers, Denmark) (Figure 2f). This was performed to ensure all samples were of the same dimensions after repairing. All the specimens were polished using a polishing machine with 400 grit sandpaper and 1000 grit sandpaper (SiC Paper, Struers, United States) to achieve the final smooth surfaces at the final desired dimension. Half of the specimens in each group were subjected to thermocycling in a 5°C and 55°C thermal cycler (VWR, Proto‐tech, USA) with a dwell time of 30 s. The thermal cycler was set to 10,000 cycles to represent 12 months of in vitro functioning (Gale and Darvell 1999). The thermocycling (TC) specimens were tested for flexural strength right after removal from the thermocycler. The non‐thermocycling (NTC) specimens were stored at room temperature in distilled water for 24 h before testing. This allowed for water to diffuse into the polymer chains and subsequent volumetric hygroscopic expansion (Alhotan et al. 2021).

### 2.3 Three‐Point Bend Test and Analyses

**Figure 3:**
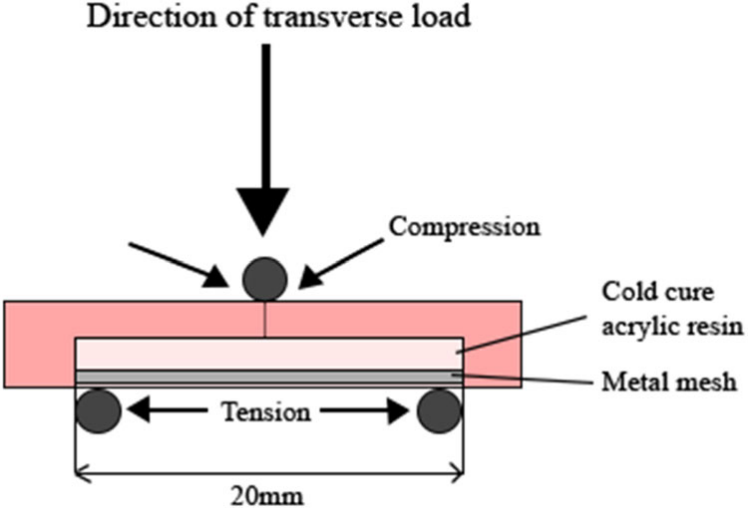
The lateral view of a three‐point bending scenario: NF—non‐primed fine stainless steel mesh, ^P^F—primed fine stainless steel mesh, NM—non‐primed med‐fine stainless steel mesh, PM—primed med‐fine stainless steel mesh, NTC—non‐thermocycling, TC—thermocycling.

Flexural strength testing was carried out with a universal testing machine (Instron 3369, Instron, USA) with a 500N load cell at a crosshead speed of 2 mm/min at a 20 mm distance (Figure 3). The specimens were loaded to the fracture point, and stress–strain curves and maximum load (*N*) values were recorded using Bluehill Universal software (Version 4.08, Instron). The flexural strength was calculated using the following equation: $\sigma =\frac{3\text{FL}}{{2\text{bh}}^{2}}$, where *σ* is flexural strength, *F* is the load at fracture (*N*), *L* is the support span distance (20 mm), *b* is the width, and *h* is the height (in mm) of the specimen (Anusavice et al. 2012). Flexural strength was further analyzed by a Weibull analysis in which failure probability can be predicted at any level of stress, providing information about the variability of the results and reflecting the structural reliability of materials or bonded parts. Weibull modulus was calculated using the following equation: $P_{f}=1-\text{exp}\left[-{\left(\frac{\sigma -\sigma_{u}}{\sigma_{\theta }}\right)}^{m}\right]$, where *P_f_* is the fracture probability of the specimen, *σ* is the fast‐fracture strength without the influence of subcritical crack growth (SCG), *σ_θ_* is the characteristic strength, and *m* is the Weibull modulus (Nemeth et al. 2005).

The results of the study were statistically analyzed using statistical software (IBM SPSS version 28.0.1.0). The mean and standard deviation values for all testing groups were reported in descriptive statistics. The flexural strength was compared among the groups using one‐way analysis of variance (ANOVA) and Tukey's tests to determine a significant difference. One represented sample from each group was selected for scanning electron microscopy (SEM) analysis to determine the mode of failure. The samples were positioned on their longitudinal side under a scanning electron microscope (JSM‐6700F, JEOL Ltd., Japan) for fractography analysis. Crack propagation and the interface of the stainless steel mesh and self‐cure acrylic resin at the fracture site were photographed at different magnifications of ×25 and ×100 at an accelerating voltage of 5.0 kV.

## 3 Results

### 3.1 Flexural Strength

**Figure 4:**
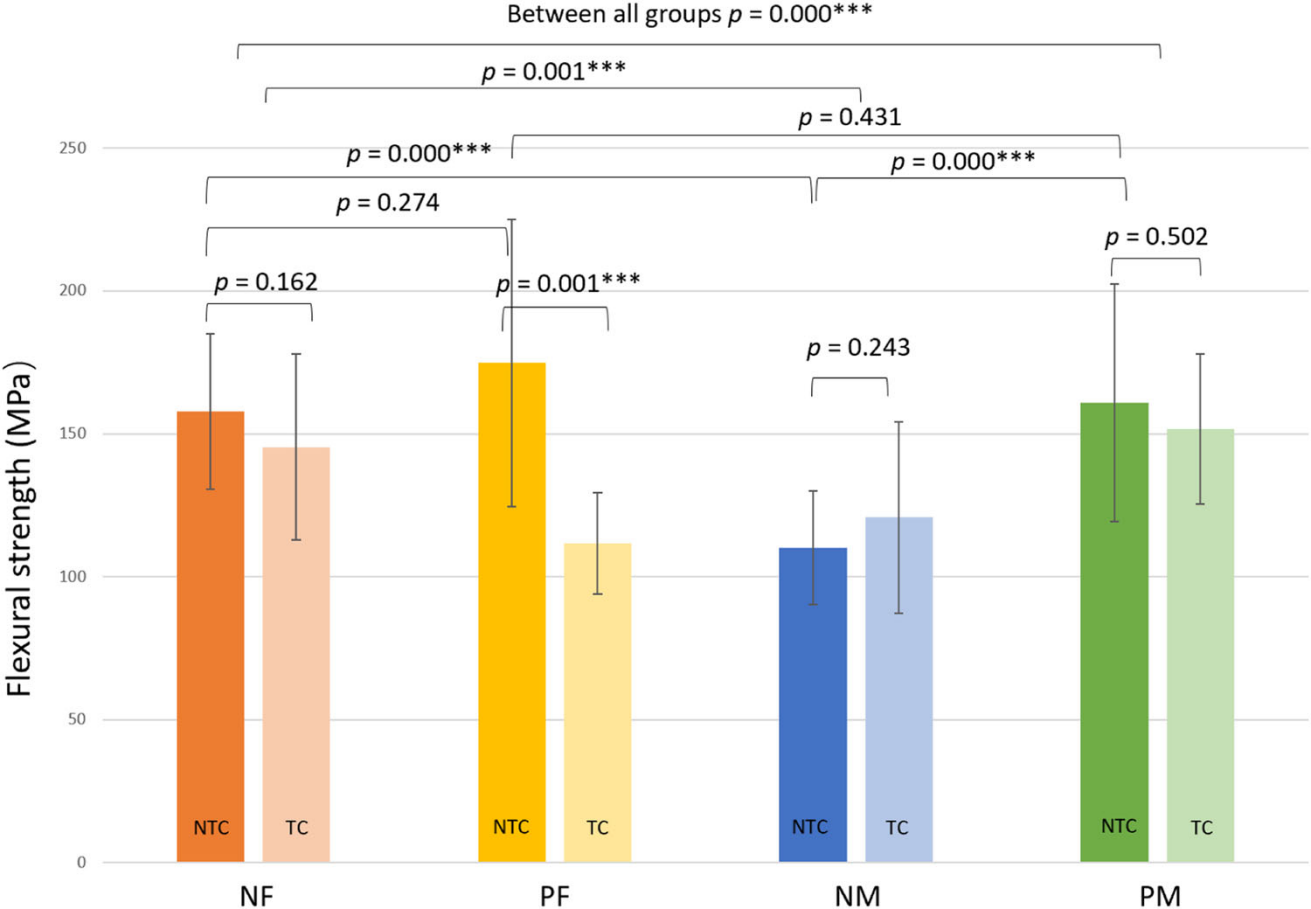
Mean flexural strengths and statistical significances of all tested groups. Vertical bars—standard deviation. Horizontal bars show *p* values between various groups. NF—non‐primed fine stainless steel mesh, PF—primed fine stainless steel mesh, NM—non‐primed med‐fine stainless steel mesh, PM—primed med‐fine stainless steel mesh, NTC—non‐thermocycling, TC—thermocycling.

The mean flexural strengths and standard deviation values of the testing groups are summarized in Table 2 and Figure 4. The highest mean flexural strength was recorded in group NTC‐PF (174.80 ± 50.27 MPa). The mean flexural strength of fine stainless steel mesh both primed (174.80 ± 50.27 MPa) and non‐primed (157.75 ± 27.14 MPa) NTC groups were higher than both groups of NTC mid‐fine stainless steel reinforcement groups.

**Table 2:** Mean ± standard deviation values of the flexural strength (MPa) for non‐thermocycle and thermocycle groups.

Groups (abbreviation for each group)	Non‐thermocycle (NTC) results Mean ± SD (MPa)	Thermocycle results (TC) Mean ± SD (MPa)
Non‐primed fine stainless steel mesh (NF)	157.75 ± 27.14	145.33 ± 17.67
Primed fine stainless steel mesh (PF)	174.80 ± 50.27	111.59 ± 33.45
Non‐primed med‐fine stainless steel mesh (NM)	110.21 ± 19.85	120.73 ± 26.35
Primed med‐fine stainless steel mesh (PM)	160.87 ± 41.50	151.79 ± 27.92

When comparisons were made among the (NTC) groups, significantly higher mean flexural strength was recorded for the group NTC‐PM (160.87 ± 41.50 MPa) than group NTC‐NM, which had a mean flexural strength of 110.21 ± 19.85 MPa (*p* < 0.05). For the nonadhesive primer application groups, a significantly higher mean flexural strength was recorded for the NTC‐NF compared to the NTC‐NM group (157.75 ± 27.14 MPa *>* 110.21 ± 19.85 MPa; *p* < 0.05). A significant reduction in mean flexural strength (*p* < 0.05) after 10,000 thermal cycles was recorded in the fine mesh group with adhesive primer application (111.59 ± 33.45 MPa). The highest mean flexural strength (151.79 ± 27.92 MPa) in the (TC) groups was recorded in the med‐fine mesh group primed with adhesive primer.

### 3.2 Weibull Analysis

**Figure 5:**
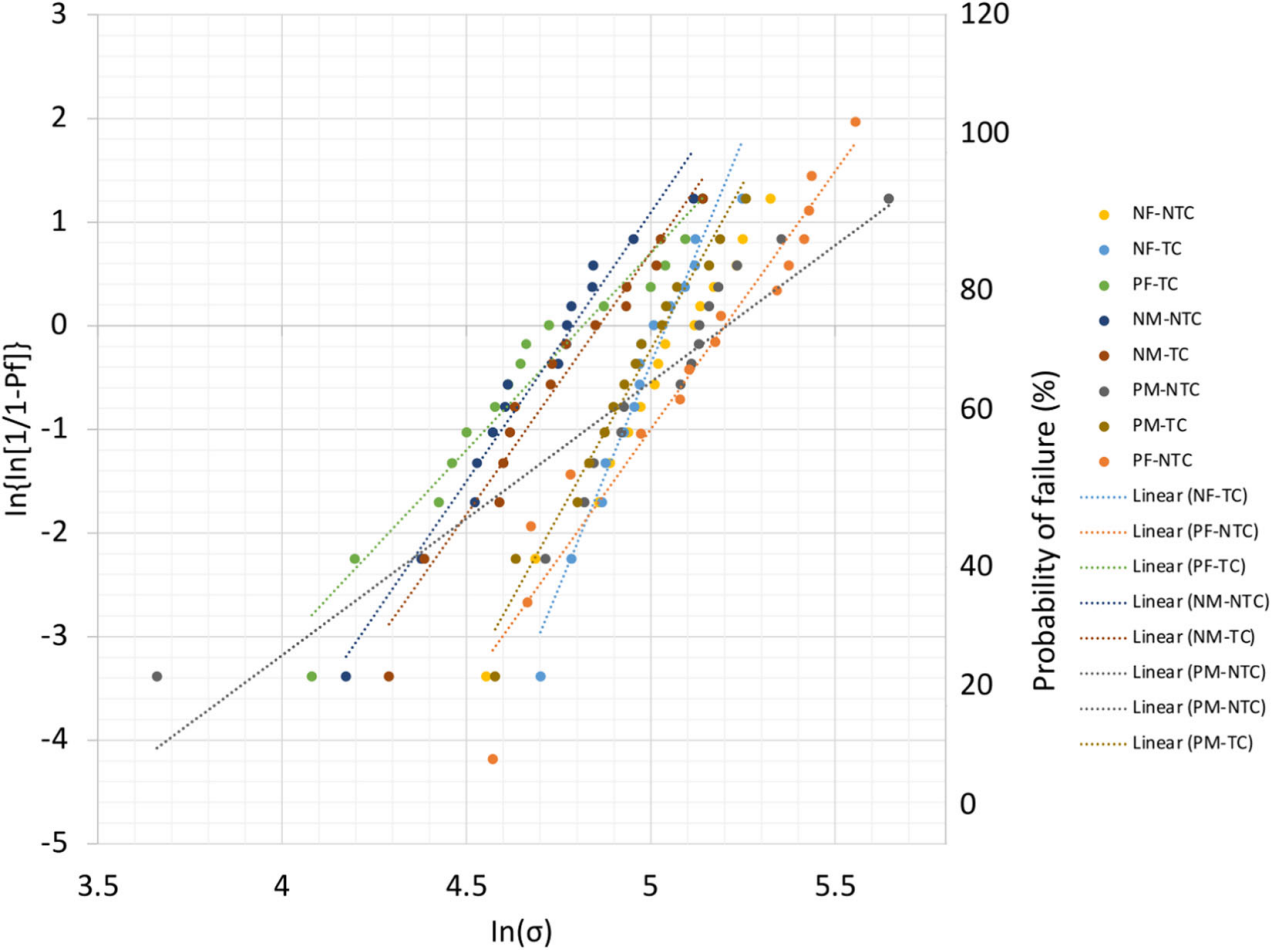
Weibull plot of all the test groups fractured during the three‐point bend test. NF—non‐primed fine stainless steel mesh, ^P^F—primed fine stainless steel mesh, NM—non‐primed med‐fine stainless steel mesh, PM—primed med‐fine stainless steel mesh, NTC, non‐thermocycling, TC—thermocycling.

Weibull modulus and normalizing strength values are presented in Table 3. For the NTC groups, group NF exhibited the highest Weibull modulus (5.86) and group PM had the lowest Weibull modulus (2.64). After thermocycling, Group NF exhibited the highest Weibull modulus (8.65) and the lowest Weibull modulus (3.80) was recorded for group PF (Figure 5).

**Table 3:** Weibull modulus and normalizing strength of the groups.

	Groups	Weibull Modulus, m	Normalizing strength, σθ (MPa)
NTC	NF	5.86	165.47
	PF	3.73	194.03
	NM	5.18	120.23
	PM	2.64	182.43
TC	NF	8.65	154.88
	PF	3.80	123.47
	NM	5.05	129.06
	PM	6.40	153.83

*Note:* NF—non‐primed fine stainless steel mesh, PF—primed fine stainless steel mesh, NM—non‐primed med‐fine stainless steel mesh, PM—primed med‐fine stainless steel mesh, NTC—non‐thermocycling, TC—thermocycling.

### 3.3 Scanning Electron Microscopy (SEM) Analysis

**Figure 6:**
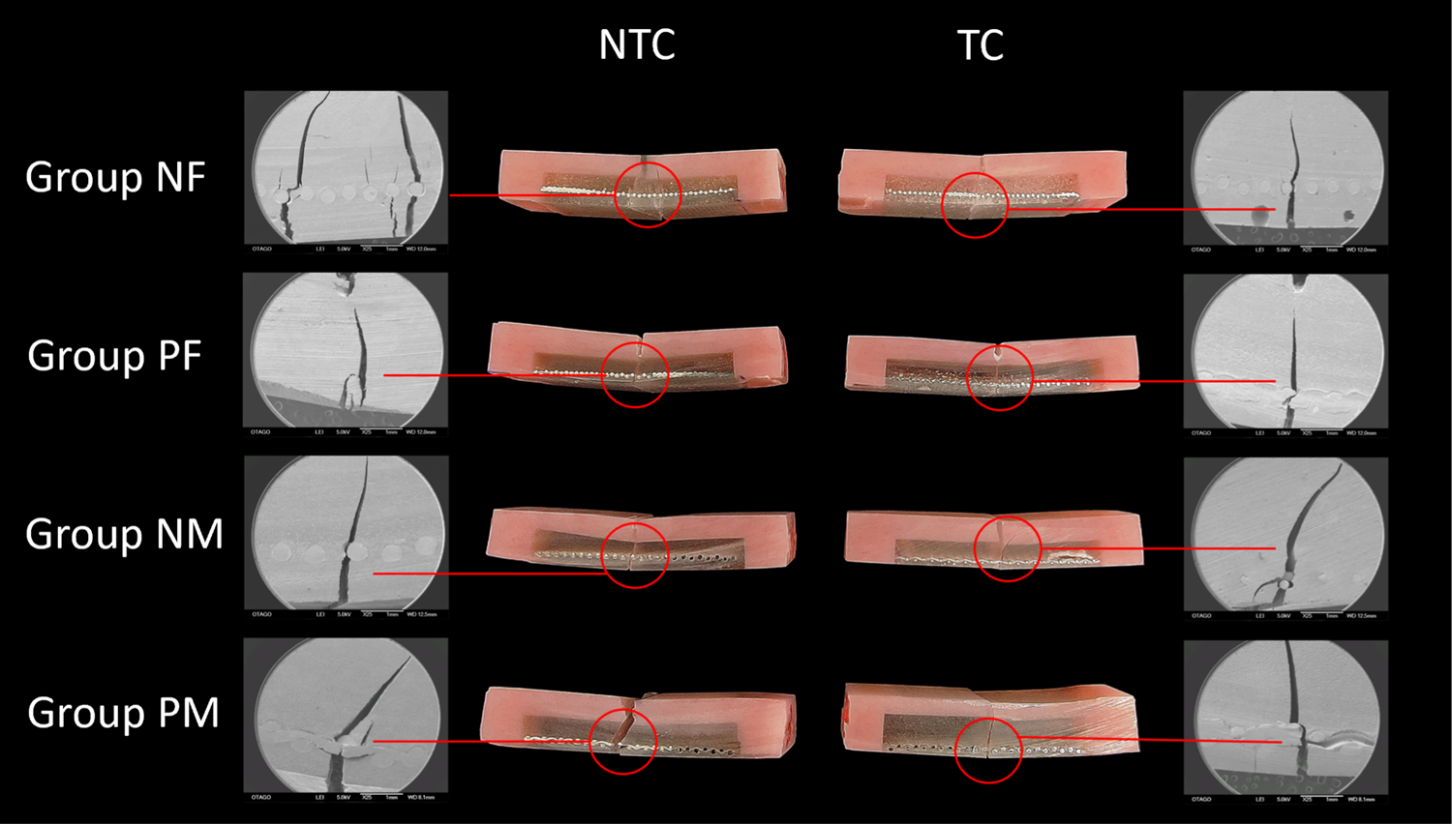
Photographs of the fractures of the selected samples from each group under the electron microscope (magnification ×25). NF—non‐primed fine stainless steel mesh, ^P^F—primed fine stainless steel mesh, NM—non‐primed med‐fine stainless steel mesh, PM—primed med‐fine stainless steel mesh, NTC—non‐thermocycling, TC—thermocycling.

**Figure 7:**
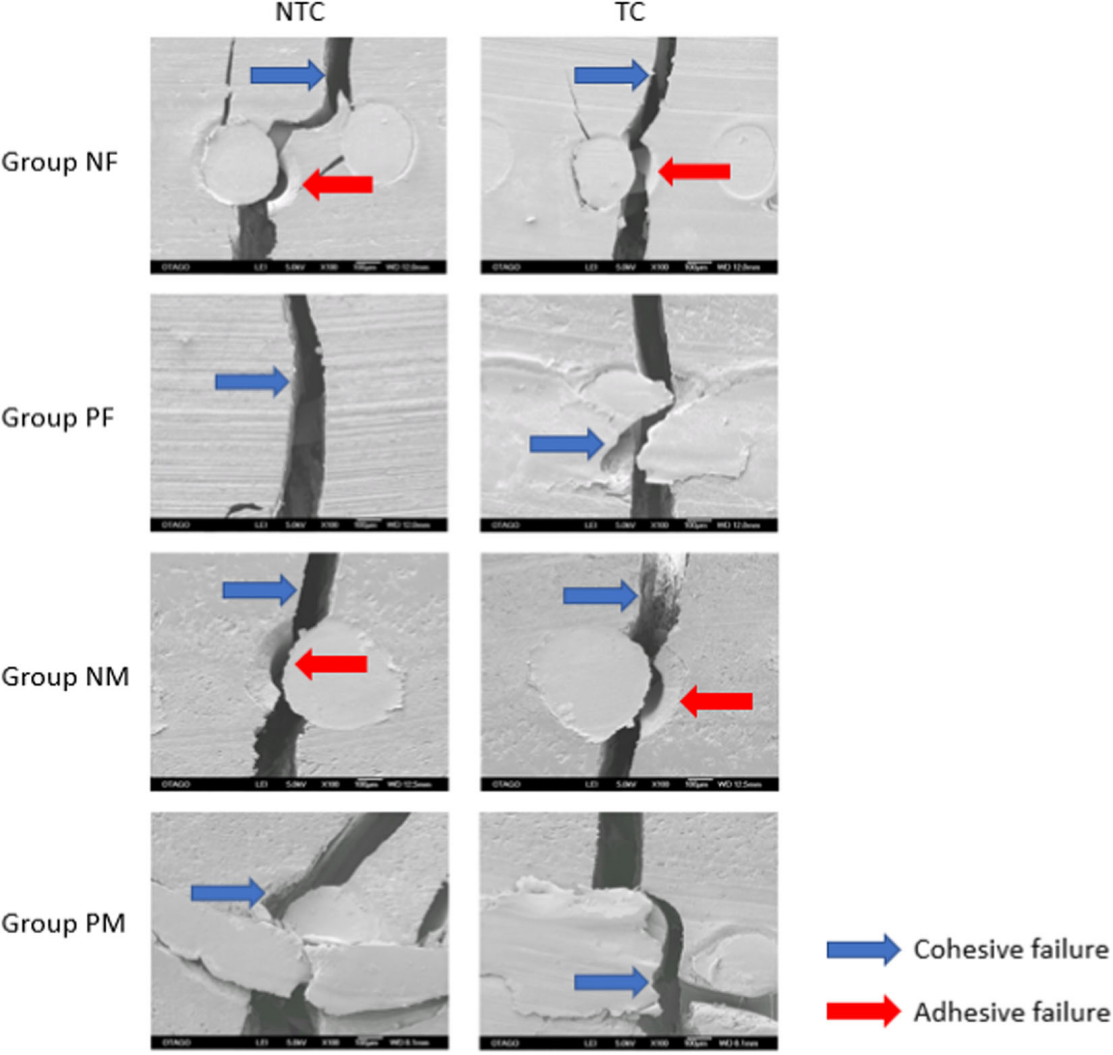
SEM images of fracture site of selected specimens from each group (magnification x100) identifing cohesive and or adhesive failure.

The lateral side of the selected specimens from each group was photographed under an electron microscope (SMZ800N, Nikon, USA), and the cracks were observed under the scanning electron microscope at a magnification of ×25 (Figure 6). The mode of failure was identified in SEM analysis with the fractures being analyzed at a higher magnification of ×100 (Figure 7). Groups that were reinforced with non‐primed stainless steel mesh had both cohesive and adhesive failures. A cohesive failure occurred within the self‐cure acrylic resin that resulted in a rough fracture interface. Adhesive failure was identified at the interface of the stainless steel mesh and the self‐cure acrylic resin for the NF and NM as indicated by red arrows in Figure 7. On the other hand, no adhesive failures were found in the groups that were repaired with primed stainless steel mesh.

## 4 Discussion

This study evaluated the influence on flexural strength of PMMA heat‐cured acrylic resin reinforced with med‐fine and fine stainless steel mesh primed with adhesive and artificial aging. The null hypothesis was rejected as there is a significant difference in the flexural strength of repaired PMMA heat‐cured acrylic resin reinforced with med‐fine and fine stainless steel mesh primed with adhesive and artificial aging.

Previous studies have reported that the incorporation of the stainless steel mesh strengthened the PMMA material after repair (Anne et al. 2017; Ardakani et al. 2021; Kumar et al. 2017). Nevertheless, these studies did not specify the position of the stainless steel mesh in the repair gap, which could be a variable and make the results inconsistent. A recent study by Li et al. (2021) reported that placing the wide (20 mm) reinforcement material (E‐glass fiber mesh) in the tension zone of the loading configuration of a three‐point bend test scenario increased the flexural strength of the PMMA specimen (Li et al. 2021). Therefore, this study ensured the position of the stainless steel mesh was located at the tension zone and further controlled the width (20 mm) and depth (3 mm) of the repair gap using the three‐axis drill press machine to standardize the method. This allowed for a direct comparison between the med‐fine and fine stainless steel meshes to be positioned with more accuracy. The study results showed that the use of fine stainless steel mesh reinforcement with adhesive primer significantly increased the mean flexural strength, while thermocycling reduced the mean flexural strength of the repaired PMMA heat‐cured acrylic resins; thus, the null hypothesis was rejected.

A significantly higher mean flexural strength was recorded in the current study in the non‐thermocycling groups of both primed and non‐primed groups with fine mesh when compared to those with med‐fine mesh. Metal mesh reinforcements have been stated to have higher modulus of elasticity, which results in the force absorption by the metal without any significant deformation of PMMA (Ardakani et al. 2021). The smaller the mesh size, the larger the surface area for the self‐cure acrylic to bond to and thus the more engagement of the material increasing the strength of PMMA. The surface area of med‐fine stainless steel mesh is two times less than the surface area of fine‐mesh stainless steel mesh, thus less surface area for the self‐cure acrylic to engage with. The amount of stainless steel alloy in the mesh may also have been a contributing factor affecting the strength. The fine stainless steel mesh has a greater amount of stainless steel alloy than the med‐fine stainless steel mesh with the same dimensions, which may have provided more rigidity when loaded. However, no statistically significant difference was found in the mean flexural strength between the fine mesh and med‐fine mesh groups with adhesive primer (*p* > 0.05), which means that the mesh size had no significant effect on the flexural strength of the specimens after priming.

The results of this study have shown that the application of the adhesive primer improved the flexural strength of repaired PMMA heat cure acrylic irrespective of the reinforcement size of mesh aperture for the non‐thermocycling groups. The Scotchbond Universal plus adhesive primer used is an acidic adhesive primer that contains a distinguishing component called methacryloyloxydecyl dihydrogen phosphate (MDP) monomer. MDP is the functional component used to develop the bond between stainless steel and self‐cure acrylic resin (Hsu et al. 2021; Ishikawa et al. 2006; Matsumura et al. 1997). An earlier study found that the MDP monomer chemically reacts to metal oxides and forms a covalent bond (Shafiei et al. 2018). The investigators further reported that the nickel and chromium metal oxides that form on the surface of the stainless steel mesh react with the MDP monomer to form chemical bonds (Shafiei et al. 2018). The adhesive primer also bonds to the self‐cure acrylic resin through the chemical component of the adhesive, namely the 2‐hydroxyethyl methacrylate (Hsu et al. 2021). The adhesive primer used in this study was therefore able to bond to both the acrylic resin and the stainless steel mesh, which may have been the contributing factor in the corresponding higher flexural strength of the adhesive primed groups. However, this significant effect of adhesive primer on the flexural strength was not observed in the non‐thermocycling fine mesh groups. Therefore, it can be assumed that the adhesive primer was more effective in improving the flexural strength of the med‐fine mesh specimens.

The mean strength values in our study for the four non‐thermocycle test groups were higher than the 12 months of in vitro thermocycling test groups and is consistent with the findings of Li et al. (2021), which also found that aging reduces the overall flexural strength. During thermocycling, PMMA acrylic specimens were subjected to 5°C and 55°C temperatures. Water molecules can diffuse into the denture base materials quicker at an increased temperature. The diffused water in the polymers acts as a plasticizer and thus allows the chains of polymers to slide under compression forces easily. Under thermocycling, the specimens are exposed to thermal stresses routinely. This causes degradation of the surface and weakens the mechanical properties of the material, thus lowering the flexural strength (Polychronakis et al. 2017). However, there was an increase in the mean flexural strength of Group NM after thermocycling. This improvement of the mechanical property may be due to further polymerization in heating and water immersion during thermocycling when the acrylic resins were not polymerized completely in the first place (Rashid et al. 2015; Kostić et al. 2020; de Silva et al. 2013). Thermocycling can simulate the thermal stresses a denture material undergoes in an oral environment (Eliasson and Dahl 2020); therefore, it is important to note the flexural strength values after thermocycling, as they will have a higher clinical significance. Although Group PF had the highest mean flexural strength before thermocycling, it had the lowest mean flexural strength at 111.59 ± 33.45 MPa after thermocycling. Group PM can therefore be identified as performing the best in a clinical situation as it had the highest mean flexural strength at 151.79 ± 27.92 MPa after thermocycling. The mean flexural strength values for the four non‐thermocycle test groups for the current study were higher than those reported in Li et al.'s (2021) study. Both studies have used the same brand of heat cure and self‐cure PMMA. The current study results confirm the findings of Anne et al.'s (2017) and Ardakani et al.'s (2021) studies that the stainless steel mesh provides better reinforcement than the fiber mesh.

To measure and determine the reliability characteristics of each testing group, a Weibull analysis was performed. The data for the control group from the study by Li et al. (2021) was utilized as the study protocols were duplicated and the control information was very applicable to this study's results. A Weibull plot was made using the data obtained from the three‐point bend test, which mapped the probability of failure at varying stresses and visually showed the relationship between each group's failure. The normalizing strength is also known as characteristic strength; *σ_θ_* is the level of strength below which 63.2% of all test results are expected to fail. The variability in the material strength of a brittle material can be measured by the Weibull modulus. Under the same testing conditions, specimens in the same group may fail differently due to the size and distribution of defects present in the specimens. When uniform defects are evenly distributed throughout the entire volume, the Weibull modulus is higher. A Weibull modulus below 5 suggests that the result of the group is not reliable due to different sizes of defects formed during the manufacturing process and the defects are not evenly distributed; thus, the material does not have high level of homogeneity and stability (Anusavice et al. 2012). The Weibull modulus affects the probability of failure at the stress values around the normalizing strength, so these two parameters were looked at together when interpreting the results in the Weibull analysis. Group PF‐NTC had the highest normalizing strength at 194.03 MPa, which means that 63.2% of the specimens from the group are expected to fail at 194.03 MPa. However, Group PF‐NTC had a Weibull modulus of 3.73. A low Weibull modulus means that the flexural strength of the material is less predictable, and thus, the failure around the normalizing strength would not be as accurate. In contrast, Group NF‐NTC had a narrow Weibull curve with a high Weibull modulus (5.86) confirming that the prediction of the probability of failure was accurate. In addition, Group NM‐NTC had a Weibull modulus above 5 despite a lower normalizing strength (120.23 MPa). Hence, the groups that were not primed had low variation and high consistency in flexural strength compared to the groups that were primed.

The SEM analysis of the mode of failure of each group showed that Group NF and Group NM had both cohesive and adhesive failures, whereas Group PF and Group PM only showed cohesive failure. The smooth fracture surfaces created at the interface of the stainless steel mesh and the self‐cure acrylic were due to adhesive primer failure. Adhesive failure was only observed in the non‐primed groups because there was no adhesive primer applied to achieve bonding. Cohesive failures occurred within the material and produced a highly rough cracked surface. The ideal mode of failure is a cohesive failure because this represents a failure within a material, typically at the maximum strength of that material (Ebnesajjad 2014). The identified mode of failure supports findings of this study, where the mean flexural strengths of the primed groups were higher than the non‐primed groups due to the bonding effect of the adhesive primer. The crack propagation under the electron microscope showed that only the sample from Group PM‐NTC experienced complete fracture while the other samples only showed cracks. Although the stainless steel mesh was still holding the acrylic resin together, it is important to repair the denture if cracks in the acrylic resin are present. Unrepaired acrylic resin fractures in a denture can create a site for food particle accumulation and plaque formation.

A limitation of the present study was that only one type of adhesive primer was used to prime the stainless steel mesh. Stainless steel mesh can also be bonded to acrylic resin with other types of adhesive primers such as UV curing, cyanoacrylate, and methyl‐methacrylic, which were not investigated in this study; therefore, these results cannot be broadly applied to other adhesive primers available in the market. In addition to the positive effect of adhesive on the flexural strength in this study, the effect of thermocycling on the flexure strength of stainless steel mesh‐reinforced PMMA heat cure acrylic resins should also be noted since the thermocycle results more accurately depict the oral environment. Future studies could investigate how that reinforcement with stainless steel affects additively manufactured material.

## 5 Conclusion

Within the limitations of this study, the results revealed that:

1. The application of Scotchbond Universal adhesive primer to both mid‐fine and fine stainless steel mesh significantly improved the flexural strength of the repaired PMMA heat cure acrylic resin compared to the repair group reinforced with non‐primed mesh.
2. Reinforcement with fine stainless steel mesh primed with Scotchbond Universal adhesive primer resulted in significantly higher flexural strength of repaired PMMA heat cure acrylic resin compared to reinforcement with primed med‐fine stainless steel mesh.
3. The use of thermal cycling to artificially age the samples resulted in a decrease in flexural strength, across all groups, except for the group reinforced with non‐primed med‐fine stainless steel mesh.

## Author Contributions

Gray Hun Li, John Aarts, and Joanne Choi contributed to writing – review and editing, writing – original draft, project administration, methodology, investigation, formal analysis, data curation, and conceptualization. Vidya Mudaliar and Andrew Cameron contributed to data curation, further analysis writing – original draft, and review and editing.

## Conflicts of Interest

The authors declare no conflicts of interest.

## Data Availability

The data that support the findings of this study are available from the corresponding author upon reasonable request.

## References

[cre270127-bib-0001] Alhareb, A. O. , H. M. Akil , and Z. A. Ahmad . 2017. “Impact Strength, Fracture Toughness and Hardness Improvement of PMMA Denture Base Through Addition of Nitrile Rubber/Ceramic Fillers.” Saudi Journal for Dental Research 8, no. 1: 26–34.

[cre270127-bib-0002] Alhotan, A. , J. Yates , S. Zidan , J. Haider , C. A. Jurado , and N. Silikas . 2021. “Behaviour of PMMA Resin Composites Incorporated With Nanoparticles or Fiber Following Prolonged Water Storage.” Nanomaterials (Basel) 11, no. 12: 3453. 34947803 10.3390/nano11123453 PMC8707186

[cre270127-bib-0003] AlQahtani, M. , and S. B. Haralur . 2020. “Influence of Different Repair Acrylic Resin and Thermocycling on the Flexural Strength of Denture Base Resin.” Medicina 56, no. 2: 50. 31973219 10.3390/medicina56020050 PMC7074266

[cre270127-bib-0004] Andreescu, C. F. , D. L. Ghergic , O. Botoaca , V. Hancu , A. M. Banateanu , and D. N. Patroi . 2018. “Evaluation of Different Materials Used for Fabrication of Complete Digital Denture.” Materiale Plastice 55, no. 1: 124–128.

[cre270127-bib-0005] Anne, G. , N. P. Mukarla , P. Manne , R. Anne , S. B. Muvva , and G. P. Krishna . 2017. “Comparative Evaluation of Flexural Strength of Conventional and Reinforced Heat Cure Acrylic Resins: An In Vitro Study.” Journal of Dental Research and Reviews 4, no. 1: 9–12.

[cre270127-bib-0006] Anusavice, K. , C. Shen , and H. R. Rawls . 2012. Philips' Science of Dental Materials (12th ed.). W. B. Saunders.: Elsevier Health Sciences.

[cre270127-bib-0007] Ardakani, Z. H. , R. Giti , S. Dabiri , A. H. Hosseini , and M. Moayedi . 2021. “Flexural Strength of Polymethyl Methacrylate Reinforced With High‐Performance Polymer and Metal Mesh.” Dental Research Journal 18, no. 30: 30. 34322206 PMC8314965

[cre270127-bib-0008] Arioli Filho, J. N. , L. E. Butignon , R. P. Pereira , M. G. Lucas , and F. A. Mollo Junior, Jr. 2011. “Flexural Strength of Acrylic Resin Repairs Processed by Different Methods: Water Bath, Microwave Energy and Chemical Polymerization.” Journal of Applied Oral Science 19, no. 3: 249–253. 21625742 10.1590/S1678-77572011000300013 PMC4234338

[cre270127-bib-0009] Beyli, M. S. , and J. A. Von Fraunhofer . 1981. “An Analysis of Causes of Fracture of Acrylic Resin Dentures.” Journal of Prosthetic Dentistry 46, no. 3: 238–241. 7021802 10.1016/0022-3913(81)90206-7

[cre270127-bib-0010] Ebnesajjad, S. 2014. “Chapter 5 ‐ Theories of Adhesion.” In Surface Treatment of Materials for Adhesive Bonding, edited by S. Ebnesajjad , Vol. 2, 77–91. William Andrew Publishing.

[cre270127-bib-0011] Eliasson, S. T. , and J. E. Dahl . 2020. “Effect of Thermal Cycling on Temperature Changes and Bond Strength in Different Test Specimens.” Biomaterial Investigations in Dentistry 7, no. 1: 16–24. 32128509 10.1080/26415275.2019.1709470 PMC7033714

[cre270127-bib-0012] Fonseca, R. B. , A. V. B. Kasuya , I. N. Favarão , L. Z. Naves , and M. G. Hoeppner . 2015. “The Influence of Polymerization Type and Reinforcement Method on Flexural Strength of Acrylic Resin.” Scientific World Journal 2015: 919142. 25879079 10.1155/2015/919142 PMC4386715

[cre270127-bib-0013] Gale, M. S. , and B. W. Darvell . 1999. “Thermal Cycling Procedures for Laboratory Testing of Dental Restorations.” Journal of Dentistry 27, no. 2: 89–99. 10071465 10.1016/s0300-5712(98)00037-2

[cre270127-bib-0014] Goracci, C. , M. Özcan , L. Franchi , G. Di Bello , C. Louca , and A. Vichi . 2019. “Bracket Bonding to Polymethylmethacrylate‐Based Materials for Computer‐Aided Design/Manufacture of Temporary Restorations: Influence of Mechanical Treatment and Chemical Treatment With Universal Adhesives.” Korean Journal of Orthodontics 49, no. 6: 404–412. 31815108 10.4041/kjod.2019.49.6.404 PMC6883210

[cre270127-bib-0015] Heidari, B. , F. Firouz , A. Izadi , S. Ahmadvand , and P. Radan . 2015. “Flexural Strength of Cold and Heat Cure Acrylic Resins Reinforced With Different Materials.” Journal of Dentistry (Tehran, Iran) 12, no. 5: 316–323. 26877726 PMC4749095

[cre270127-bib-0016] Hsu, C.‐T. , J.‐H. Tsai , T.‐M. Huang , et al. 2021. “Atmospheric Pressure Plasma Jet Treatment Enhances the Effect of Alloy Primer on the Bond Strength Between Polymethyl Methacrylate and Stainless Steels: Application for Retention of Magnetic Attachment to Resin Denture Base.” Colloids and Surfaces B: Biointerfaces 197: 111440. 33130522 10.1016/j.colsurfb.2020.111440

[cre270127-bib-0017] Ishikawa, Y. , Y. Kawamoto , H. Koizumi , M. Furuchi , H. Matsumura , and N. Tanoue . 2006. “Effect of Metal Priming Agents on Bonding Characteristics of an Acrylic Resin Joined to SUS XM27 Steel.” Journal of Oral Science 48, no. 4: 215–218. 17220619 10.2334/josnusd.48.215

[cre270127-bib-0018] Kalra, S. , V. Kharsan , and N. Kalra . 2015. “Comparative Evaluation of Effect of Metal Primer and Sandblasting on the Shear Bond Strength Between Heat Cured Acrylic Denture Base Resin and Cobalt‐Chromium Alloy: An In Vitro Study.” Contemporary Clinical Dentistry 6, no. 3: 386–391. 26321840 10.4103/0976-237X.161895 PMC4549992

[cre270127-bib-0019] Kim, S. S. , M. S. Vang , H. S. Yang , S. W. Park , and H. P. Lim . 2009. “Effect of Adhesive Primers on Bonding Strength of Heat Cure Denture Base Resin to Cast Titanium and Cobalt‐Chromium Alloy.” Journal of Advanced Prosthodontics 1, no. 1: 41–46. 21165254 10.4047/jap.2009.1.1.41 PMC2994673

[cre270127-bib-0020] Kostić, M. , J. Stanojević , A. Tačić , et al. 2020. “Determination of Residual Monomer Content in Dental Acrylic Polymers and Effect After Tissues Implantation.” Biotechnology & Biotechnological Equipment 34, no. 1: 254–263.

[cre270127-bib-0021] Kumar, V. , L. Kumar , K. Sehgal , K. Datta , and B. Pal . 2017. “A Comparative Evaluation of Effect of Reinforced Autopolymerizing Resin on the Flexural Strength of Repaired Heat‐Polymerized Denture Base Resin Before and After Thermocycling.” Journal of International Society of Preventive and Community Dentistry 7, no. Suppl 2: 99. 10.4103/jispcd.JISPCD_276_17 PMC5682712 29184836

[cre270127-bib-0022] Li, G. H. , S. Chen , A. Grymak , J. N. Waddell , J. J. Kim , and J. J. E. Choi . 2021. “Fibre‐Reinforced and Repaired PMMA Denture Base Resin: Effect of Placement on the Flexural Strength and Load‐Bearing Capacity.” Journal of the Mechanical Behavior of Biomedical Materials 124: 104828. 34530303 10.1016/j.jmbbm.2021.104828

[cre270127-bib-0023] Matsumura, H. , T. Tanaka , and M. Atsuta . 1997. “Effect of Acidic Primers on Bonding Between Stainless Steel and Auto‐Polymerizing Methacrylic Resins.” Journal of Dentistry 25, no. 3: 285–290. 9175359 10.1016/s0300-5712(96)00023-1

[cre270127-bib-0024] Minami, H. , S. Suzuki , H. Kurashige , Y. Minesaki , and T. Tanaka . 2005. “Flexural Strengths of Denture Base Resin Repaired With Autopolymerizing Resin and Reinforcements After Thermocycle Stressing.” Journal of Prosthodontics 14, no. 1: 12–18. 15733130 10.1111/j.1532-849X.2005.00006.x

[cre270127-bib-0025] Multigner, M. , E. Frutos , J. L. González‐Carrasco , J. A. Jiménez , P. Marín , and J. Ibáñez . 2009. “Influence of the Sandblasting on the Subsurface Microstructure of 316LVM Stainless Steel: Implications on the Magnetic and Mechanical Properties.” Materials Science and Engineering: C 29, no. 4: 1357–1360.

[cre270127-bib-0026] Nagai, E. , K. Otani , Y. Satoh , and S. Suzuki . 2001. “Repair of Denture Base Resin Using Woven Metal and Glass Fiber: Effect of Methylene Chloride Pretreatment.” Journal of Prosthetic Dentistry 85, no. 5: 496–500. 11357077 10.1067/mpr.2001.115183

[cre270127-bib-0027] Nemeth, N. , J. Gyekenyesi , and O. Jadaan . 2005. Inventors Lifetime Reliability Prediction of Ceramic Structures Under Transient Thermomechanical Loads. Patent NASA/TP‐2005‐212505.

[cre270127-bib-0028] Polychronakis, N. , A. Sarafianou , A. Zissis , and T. Papadopoulos . 2017. “The Influence of Thermocycling on the Flexural Strength of a Polyamide Denture Base Material.” Acta Stomatologica Croatica 51, no. 4: 309–315. 29872236 10.15644/asc51/4/5 PMC5975457

[cre270127-bib-0029] Polyzois, G. L. , P. A. Tarantili , M. J. Frangou , and A. G. Andreopoulos . 2001. “Fracture Force, Deflection at Fracture, and Toughness of Repaired Denture Resin Subjected to Microwave Polymerization or Reinforced With Wire or Glass Fiber.” Journal of Prosthetic Dentistry 86, no. 6: 613–619. 11753313 10.1067/mpr.2001.120069

[cre270127-bib-0030] Rached, R. N. , J. M. Powers , and A. A. Del Bel Cury . 2004. “Repair Strength of Autopolymerizing, Microwave, and Conventional Heat‐Polymerized Acrylic Resins.” Journal of Prosthetic Dentistry 92, no. 1: 79–82. 15232565 10.1016/j.prosdent.2004.04.005

[cre270127-bib-0031] Rashid, H. , Z. Sheikh , and F. Vohra . 2015. “Allergic Effects of the Residual Monomer Used in Denture Base Acrylic Resins.” European Journal of Dentistry 09, no. 4: 614–619. 10.4103/1305-7456.172621 PMC4745248 26929705

[cre270127-bib-0032] Seó, R. S. , K. H. Neppelenbroek , and J. N. A. Filho . 2007. “Factors Affecting the Strength of Denture Repairs.” Journal of Prosthodontics 16, no. 4: 302–310. 17451476 10.1111/j.1532-849X.2007.00191.x

[cre270127-bib-0033] Shafiei, F. , M. Behroozibakhsh , A. Abbasian , and S. Shahnavazi . 2018. “Bond Strength of Self‐Adhesive Resin Cement to Base Metal Alloys Having Different Surface Treatments.” Dental Research Journal 15, no. 1: 63–70. 29497449 10.4103/1735-3327.223610 PMC5806432

[cre270127-bib-0034] de Silva, S. , A. L. Machado , A. Chaves Cde , A. C. Pavarina , and C. E. Vergani . 2013. “Effect of Thermal Cycling on Denture Base and Autopolymerizing Reline Resins.” Journal of Applied Oral Science: Revista FOB 21, no. 3: 219–224. 23857648 10.1590/1679-775720130061 PMC3881901

[cre270127-bib-0035] Somani, M. , M. Khandelwal , V. Punia , and V. Sharma . 2019. “The Effect of Incorporating Various Reinforcement Materials on Flexural Strength and Impact Strength of Polymethylmethacrylate: A Meta‐Analysis.” Journal of Indian Prosthodontic Society 19, no. 2: 101–112. 31040543 10.4103/jips.jips_313_18 PMC6482623

[cre270127-bib-0036] Taylor, M. , M. Masood , and G. Mnatzaganian . 2021. “Longevity of Complete Dentures: A Systematic Review and Meta‐Analysis.” Journal of Prosthetic Dentistry 125, no. 4: 611–619. 32359852 10.1016/j.prosdent.2020.02.019

